# Ring‐specific vulnerability to embolism reveals accumulation of damage in the xylem

**DOI:** 10.1111/nph.70137

**Published:** 2025-04-18

**Authors:** Jaycie C. Fickle, German Vargas G., William R. L. Anderegg

**Affiliations:** ^1^ School of Biological Sciences University of Utah Salt Lake City UT 84112 USA; ^2^ Department of Botany and Plant Pathology Oregon State University Corvallis OR 97331 USA; ^3^ Department of Forest Ecosystems and Society Oregon State University Corvallis OR 97331 USA; ^4^ Wilkes Center for Climate Science and Policy University of Utah Salt Lake City UT 84112 USA

**Keywords:** Aspen, climate change, embolism, hydraulic conductivity, ring‐specific hydraulic conductivity

## Abstract

- Human‐caused climate change is predicted to bring more frequent droughts and higher temperatures in the western United States, which threaten ecologically important trembling aspen forests.
- We used ring‐specific vulnerability curves of aspen branches along two climate gradients to determine whether damages to pit membranes accumulate as the xylem ages.
- We found that rings older than 3 yr have a significant decline in hydraulic conductivity, especially at average summer water potentials for the species. These differences were not due to differences in the diameter of the vessels, but a difference in how much xylem was active between rings older than 3 yr and 1 yr, suggesting the presence of accumulated damage to pit membranes impairing water transport.
- Vulnerability to embolism differs across ring age and between wetter and drier populations, underscoring that damages due to drought may accumulate to lethal levels if the xylem does not acclimate to climate change in newer growth.

## Introduction

Forests play an important role in the Earth system, as they impact carbon, water, and energy cycles as well as the climate (Bonan, 2008). However, forests have been threatened due to anthropogenic climate change, which has brought more frequent drought events alongside increasing atmospheric moisture demand due to higher temperatures (Williams *et al*., 2013; IPCC, 2014; Spinoni *et al*., 2018; Grossiord *et al*., 2020). These climate change‐type droughts have resulted in damages to the xylem that limit tree growth for many years after drought or lead to mortality events (Breshears *et al*., 2005; Anderegg *et al*., 2013b, 2015), which threaten forests' role as a carbon sink (Allen *et al*., 2010). Climate change‐driven changes in drought regimes highlight the need to understand the long‐term impacts of drought stress on plant hydraulic transport. Particularly, whether damages accumulate to lethal levels or whether xylem can potentially recover and even shift through new growth to become acclimated and more resistant to drought is a crucial unknown (Corcuera *et al*., 2004; Jacobsen & Pratt, 2014; Tomasella *et al*., 2019; Anderegg *et al*., 2020; Pratt *et al*., 2020; Pritzkow *et al*., 2021).

The physiology of xylem responses to multiple droughts is complex and poorly understood. Severe drought events that cause continuous exposure to drought stress may induce xylem damages that increase its vulnerability to drought, (i.e. cavitation fatigue) in some species (Hacke *et al*., 2001; Feng *et al*., 2021). During drought stress, xylem tensions may exceed the tolerances of conduits as it impacts the membranes within their pits. When this occurs, gasses are pulled into conduits and create emboli, which block the ability of conduits to transport water. Emboli can spread from one conduit to another through pit membranes. Cavitation fatigue occurs when permanent damage on pit membranes takes place and they become less resistant to air seeding (i.e. ‘leaky’ pits), even if the conduits are refilled (Hacke *et al*., 2001). Furthermore, xylem aging can also impact embolism resistance and water conductance over long periods of time (Sperry *et al*., 1991). Remarkably, to our knowledge, there has not been a lot of work on xylem aging and changes in vulnerability in individual rings over time, as almost all studies examine the response of a full stem or in large sections of bole xylem (Domec & Gartner, 2001; Spicer & Gartner, 2001; Delzon *et al*., 2004; Ford *et al*., 2004; but see Melcher *et al*., 2003, for an example study using vessel‐specific vulnerability patterns, Domec & Gartner, 2002, for patterns between the earlywood and latewood, and Venturas *et al*., 2019, for comparisons between primary and secondary xylem), and thus, variations in vulnerability and conductivity across specific rings are not well‐understood. Prior comparison across different aged stems did result in variable conductivity, but these patterns were found in a ring‐porous species and might vary across differing wood anatomy types (Pratt *et al*., 2020).

The western United States has experienced multiple severe ‘climate change‐type’ droughts in the past several decades, which makes it an ideal place to examine the responses of plant hydraulics to multiple droughts (Breshears *et al*., 2005; Williams *et al*., 2013; Zhang *et al*., 2021). These severe droughts have impacted multiple dominant tree species, including ecologically important trembling aspen (*Populus tremuloides* Michx.) forests. In the past 15 yr, aspen forests have been undergoing a widespread mortality event known as sudden aspen decline (SAD; Worrall *et al*., 2008; Anderegg *et al*., 2013a,b). The SAD appears to be associated with multiyear accumulated damages in the xylem due to cavitation fatigue (Anderegg *et al*., 2013b; Feng *et al*., 2021). Forests that have experienced SAD may be more at risk in the future to less severe droughts (Anderegg *et al*., 2013b), a pattern that is often seen in other woody angiosperms exposed to drought stress (DeSoto *et al*., 2020). Additionally, freeze–thaw embolism may also interact with drought stress to increase vulnerability of the xylem (Sperry *et al*., 1994; Langan *et al*., 1997; Feng *et al*., 2015). Freezing events can increase the background tension put on the water transporting system, leading to additional ‘frost fatigue’ (Feng *et al*., 2015). The size of the conduits impacts the susceptibility of both freeze–thaw embolism and drought embolism and may therefore be useful in indicating differences in conductivity and vulnerability to both types of xylem stressors (Langan *et al*., 1997).

We tested whether xylem exhibited the accumulation of damage from multiple severe droughts in aspen forests in the western United States. We aimed to answer three main questions using hydraulic and anatomical data along multiple climate gradients: (1) How do different age rings differ in their ability to resist embolism? We hypothesize that older rings will be more vulnerable to embolism; (2) how do different aged rings differ in their hydraulic conductivity and area of active xylem area? We hypothesize that newer rings will have more conductivity and a higher percentage of active conductive area; (3) how do aspen populations from different drought regimes differ in their accumulation of damage? We hypothesize that aspen from areas with a more severe drought regime will experience increased cavitation fatigue and thus increased vulnerability to embolism.

## Materials and Methods

### Overview

We used hydraulic vulnerability curves to quantify the xylem's vulnerability to embolism in *Populus tremuloides* (Michx.). Most vulnerability curves are constructed using the entire cross‐sectional area of a sample; however, these curves do not consider differences among annual growth rings and may be overestimating the contribution of old rings to whole stem conductance (Melcher *et al*., 2003; Fukuda *et al*., 2015). It is likely that the sharp drop early on in a vulnerability curve is due to embolism in older rings (Fukuda *et al*., 2015). To test the questions described previously, we performed ring‐specific vulnerability curves and we paired with anatomical measurements and dye perfusions to determine the functional regions of xylem in aspen populations across two climate gradients.

### Study system

We used two complementary climate gradients: an elevational climate gradient (within population) and a cross‐population climatic gradient – to explore our major study questions. The elevational gradient is in Southwestern Colorado within the San Juan National Forest (SJNF). We used south‐facing forest stands situated at three elevation bands (low, mid, and high) that span a range of elevations from 2600 m to 3200 m, separated every 300 m (Anderegg & Hillerislambers, 2016). Along this gradient, precipitation increases and temperature decreases with elevation (Supporting Information Tables S1, S2). We collected samples from the San Juan elevation gradient from three elevation bands on 8 July and 23 August 2021. The proximity of the aspen stands in the SJNF allowed us to compare likely within the same population with high levels of gene flow.

We used a multipopulation climatic gradient to compare across different genetic populations (Kerr *et al*., 2023). We collected samples from five aspen populations spanning a climatic gradient in the Dixie, San Juan, Uncompahgre, White River, and Uinta National Forests in Colorado and Utah, USA, within the months of June to August 2022 (Table S3). We ranked sites in the order listed above as they represent the gradient from southmost to northmost and grade from hot and dry to warm and wet (Table S3). All plots had the same slope, aspect (South), and elevation (*c*. 3000 m) as described in Kerr *et al*. (2023), except for the White River site, which could not be accessed due to road conditions. Instead, we set up new White River plots slightly lower in elevation (*c*. 2665 m) with a Northeast aspect. The combination of these two gradients provided complementary tests of our questions and allowed us to look at the potential role of population‐level (i.e. genetic) differences in acclimation and accumulation patterns. The fixation index values (*F*_st_) for the climatic gradient ranged from 0.04 to 0.12 as determined by Kerr *et al*. (2023), while a single genetic population from the elevational gradient was assumed (*F*_st_ = 0).

### Sample collection and measurements

In all sites, we collected forked branches from the sun‐exposed mid‐canopy using a 20‐gauge shotgun or pole pruners (*n* = 9 for each site along the elevational gradient and *n* = 6 for each site along the climatic gradient). We immediately wrapped cut ends with damp paper towels, sealed branches in plastic bags, and placed bagged branches on ice for storage during travel back to the laboratory. The next day in the laboratory, we separated the two sides of the forked branch and removed at least 5 cm of tissue from the initial cut end using a razor blade.

**Fig. 1:**
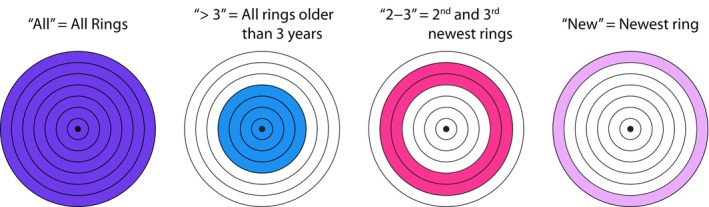
Bins in which the hydraulic measurements were made on trembling aspen branches. All figures depicting bin‐related data will match the bin names and the colors illustrated above.

One side of each forked branch was used for hydraulic measurements. Using a razor blade underwater, we gradually cut 5‐ to 10‐mm‐diameter branches down to 14 cm in length. We directly measured native hydraulic conductivity (*K*_native_) using a hydraulic apparatus as described in Sperry *et al*. (1988a), with a degassed, 0.2 μm of filtered, 10 mM KCl solution. Conductivity (*K*_h_) was measured for the entire cross‐sectional area (all), rings older than 3 yr (> 3), 2‐ to 3‐yr‐old rings (2–3), and the newest ring (new; Fig. 1) using Eqn 1:

(Eqn 1) $$K_{h}=\left(\frac{\text{Flow rate}\;\left(\frac{\mathrm{kg}}{s}\right)}{\Delta \Psi \;\left(\mathrm{MPa}\right)}\right)\cdot \text{length}\left(\mathrm{mm}\right)$$

where ΔΨ is the pressure difference between the supply of water to the stem and the height of the water level on the scale. Additionally, the temperature of the KCl solution was corrected to resistances of 20°C (Yang & Tyree, 1993). We used toothpicks to apply cyanoacrylate glue (Krazy Glue All‐Purpose glue; Newell Office Brands, Atlanta, Georgia, USA) to block off specific rings and calculated the xylem area‐specific conductivity (*K*_s_) by dividing the *K*_h_ by the area for each ring (Fig. S1). The use of cyanoacrylate glue has been shown as an effective method to block off water conductance in prior studies (Rodriguez‐Zacarro *et al*., 2019). Glue was applied to one cut end within a few seconds and then dipped in water before gluing the other side. After collecting native measurements, we trimmed superglue off both ends and submerged samples in 10 mmol of the KCl solution in a vacuum chamber overnight to remove any existing emboli. After vacuum infiltration, we remeasured the maximum hydraulic conductivity (*K*_max_) on each of the bins of rings using the same gluing technique as described above. We did these ring‐specific measurements on the same stems using this glue technique to reduce variation across different stems and avoid biases that may arise in the analyses due to using different stems (Hacke *et al*., 2022).

Vulnerability curves can be used to efficiently quantify xylem embolism resistance, as described in Tobin *et al*. (2013) and Venturas *et al*. (2016). To construct ring‐specific vulnerability curves, our samples were spun in a centrifuge at speeds correlating to the following negative pressures: −0.5, −1, −2, −4, −8, −10 MPa, or until the stems reached at least a 90% reduction in conductivity from the maximum. Before spinning in the centrifuge, we trimmed the glue off both ends with a razor blade. After centrifugation, we re‐glued ring bins and measured hydraulic conductivity for each bin of rings. We calculated the percent loss in conductivity (PLC) for each bin in each sample relative to the conductivity at −0.5 MPa (fatigue correction; Hacke *et al*., 2001; Eqn 2).

(Eqn 2) $$\mathrm{PLC}\;\left(\%\right)=\left(\frac{K_{\max}-K_{h}}{K_{\max}}\right)\times 100$$

We then used PLC data to construct vulnerability curves for each bin of rings and each population and site in the elevational gradient, removing curves with negative PLC values from the analyses. The pressure at 50% loss in conductivity (*P*_50_) was calculated by fitting each vulnerability curve to a two‐parameter Weibull fit curve (Hacke *et al*., 2015).

After completing xylem vulnerability measurements, we used Teflon‐coated razor blades (GEM single‐edge stainless steel Teflon‐coated blades; Electron Microscopy Sciences, Haffield, PA, USA) to make thin branch cross‐sections in the bottom 2 cm of the hydraulic sample. We imaged cross‐sections with a dissecting microscope (AmScope Outlet 7X‐45X Trinocular Stereo Zoom Microscope, with attached Microscope Digital Camera MU500; AmScope, Irvine, CA, USA), and measured ring area with the imagej analysis software (Schneider *et al*., 2012) or with the amscope analysis software (v.3.7.7934; AmScope). We divided conductivity by this ring area to calculate xylem area‐specific hydraulic conductivity (*K*_s_).

The other side of the collected forked branch was used for dye perfusions to determine the percentage of active xylem area. We trimmed 5‐ to 10‐mm‐diameter sections underwater to at least 10 cm in length using razor blades. Following the active dye staining protocol outlined in Jacobsen *et al*. (2007), we pulled a 2.0 pH 0.5% weight/volume Safranin O dye under *c*. −2 kPa of pressure into branch segments for 10 min or longer until dye was seen coming through the distal cut end, which were then immediately bagged and placed in a freezer until further sectioning. We used Teflon‐coated razor blades to make sections perpendicular to the branch at 5 cm from the proximal cut end and immediately imaged sections under a dissecting microscope. Using imagej, we measured the xylem area corresponding to each bin, as well as the total area of dye for each bin, allowing us to then calculate the percent area of active xylem for each bin of rings.

In order to determine whether conductivity differences among rings and populations were driven by vessel diameter sizes, we measured vessel diameter and hydraulic diameter from each bin and each population in the climatic gradient. These were measured on the same samples that hydraulic conductivity measurements were made on for the climatic gradient. We used Teflon‐coated razor blades to make thin cross‐sections that we mounted on glass slides with glycerol. We took images with a camera (7.2 Color Mosaic Camera; SPOT Imaging, Sterling Heights, MI, USA) mounted on a microscope (Nikon Eclipse E600W, Model RT KE; Diagnostic Instruments, Salt Lake City, UT, USA) at 200× magnification, and measured the area of at least 100 vessels for each bin using the imagej software. We converted vessel area to vessel diameter (with the assumption that the vessels were a circle). We also calculated the hydraulic diameter using Eqn 3.

(Eqn 3) $$D_{h}=\frac{\sum \;{\text{Vessel Diameters}}^{5}}{\sum \;{\text{Vessel Diameters}}^{4}}$$

We collected additional branches from the same trees in the Uinta (*n* = 5) and White River (*n* = 6) populations in summer 2022 to test whether radial movement of water across rings was present. This was done to determine whether blocking off water transport with glue would be successful or whether the water would just move across ring boundaries and around the glue blockages. We applied cyanoacrylate glue to the proximal end of each section covering all but the three newest rings, then carried out dye perfusions as described above. We used Teflon‐coated razor blades to section frozen samples, keeping the section with the glue applied, a section directly under the applied glue, and a section 5 cm up from the proximal end. We were careful to keep the cut sections in the same orientation as to easily line up the xylem. These sections were placed on a glass slide and mounted with glycerol, immediately imaged with a dissecting microscope, and analyzed using the imagej software. For image analysis, we identified a wedge of xylem that was clear, easy to distinguish how many rings were dyed, and easily able to be lined up for all sections. For the 5‐cm section above the proximal end, we measured the total area of xylem from rings that were not glued, in addition to the area of dye that extended past the glued region. Dye ‘leakage’ (i.e. radial movement of dye into glued rings) was quantified as a percentage of the total glued area.

In order to understand whether xylem blockages were primarily emboli or permanent blockages, we performed a dye test on paired, vacuum‐infiltrated, native spun branches. We collected branches for this dye test on 23 August 2021, from the lowest and highest elevation bands in the SJNF (*n* = 3 for each elevation band). Two branches were collected from each tree, and two segments from each branch were sectioned off and paired. For one branch, we dyed one section with Safranin O Dye under native conditions, while the other part of the branch was vacuum infiltrated with 10 mmol of KCl solution overnight, then dyed. For the second branch, we vacuum‐infiltrated both sections overnight, and dyed one after flushing, while the other was spun in a centrifuge at −2 MPa for 6 min before staining. Each dye section was at least 6 cm long, with centrifuged samples at least 10 cm long to fit in the rotor (with custom spacers for a 14‐cm rotor). We sectioned stems with a Teflon‐coated razor blade at 5 cm up from the proximal end of each section and measured stained and total xylem area with the amscope analysis software, which we then used to calculate the percentage of stained (active) xylem for each section. If there was not a difference in the percentage of active xylem between ‘spun’ and ‘flushed’ samples, we would interpret that as a permanent blockage; however, if there were differences, we would interpret that as embolism.

We obtained Palmer Severity Drought Index (PDSI) and Climatic Water Deficit (CWD) data from TerraClim (Abatzoglu *et al*., 2018; accessed 21 February 2023 and 24 February 2023, respectively) for aspen sites along the climatic gradient from 01 January 2000 to 31 December 2022 (Fig. S2). The minimum PDSI and maximum CWD for the years 2018, 2020, and 2021 were extracted for the locations of each aspen population (Table S4). We obtained the 30‐yr normals data for temperature and precipitation from PRISM (PRISM Climate Group, Oregon State University, n.d.), http://prism.oregonstate.edu, accessed 26 October 2024) for the period from January 1991 to September 2020. We counted the number of days below freezing from each year from the PRISM daily temperature minimum from 01 January 1991 to 31 December 2020 to explore potential effects of freeze–thaw embolism on xylem anatomy patterns.

### Statistical analyses

For all analyses, we used type III sum of squares in ANOVA to test for differences in bins of rings and across populations/sites and did pairwise comparisons with emmeans (Lenth, 2024) unless otherwise noted. If interaction effects were not significant, they were removed from analysis and the ANOVA was rerun (Zuur *et al*., 2009). Unless otherwise stated, data met the assumptions of the model. We conducted all statistical analyses using the R software for statistical computing version R 4.2.2 (R core team, 2022).

To test whether different aged rings and the different populations and sites differ in their ability to resist embolism, we compared values of *P*_50_ and the PLC at −1 and −2 MPa. We did this for both the climatic gradient and the elevation gradient. The PLC at −1 and −2 was compared because these are the negative pressures that aspens typically operate between (L. D. L. Anderegg *et al*., 2013; Love *et al*., 2019; Fig. S3). To do this, we fitted a linear model with the metric of PLC as the response variable and the population and bin as factors. For *P*_50_, we initially removed outliers using the interquartile method, but ultimately selected a cutoff at −4 MPa for outliers, as this is the minimum *P*_50_ for aspen found in Love *et al*. (2019) for aspen populations in the same region. For a sensitivity analysis using a −3.5 MPa cutoff for outliers, see the Fig. S4(a). We log‐transformed the data and then fitted a linear model with the metric of embolism resistance as the response variable and the population and bin of rings as factors for the climatic gradient, and elevation as a factor for the elevational gradient.

To test whether different aged rings and the different populations and sites differ in their ability to transport water, we compared the *K*_s_ at −1 and −2 MPa. To do this, we fitted a linear model with the metric of conductivity as the response variable and the population and bin as factors. In addition to comparing the *K*_s_, we also compared the percentage of each ring that actively contributed to water transport using dye perfusions. We fit a linear model with the percentage of active xylem as the response variable and population and bin as factors.

We then performed a series of tests to understand the observed patterns in the context of the climatic gradient. First, we tested whether the differences in *K*_s_ and PLC among the bins and the populations were due to vessel diameter differences. To do this, we used population and bin as factors to predict vessel diameter with an ANOVA (aov command in R). This same step was repeated with the hydraulic diameter (*D*_h_). Next, we used a type II ANOVA to determine differences in the number of days below freezing per year for each population. Then, to determine whether vessel diameter and days below freezing were correlated, we did a Spearman's rank correlation between average vessel diameter and the average number of days below freezing in the populations.

To compare the percentage of active xylem in its native state, spun state, and flushed state, a three‐way ANOVA was performed among the elevation band the branches were collected from, the state in which the stem was dyed (native, flushed, or spun), and the bin of rings.

## Results

### Elevational climate gradient

#### Ring‐specific vulnerability curves among elevations

**Fig. 2:**
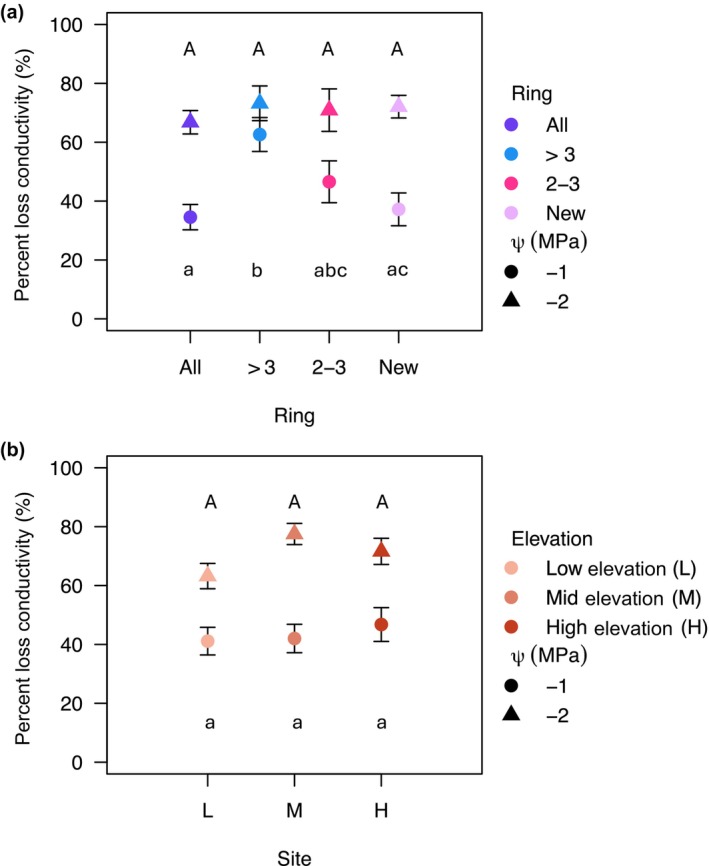
Trembling aspen percent loss in conductivity (PLC) data for the bins of rings and the three elevation bands, where the point is the mean, and the error bars represent the SE. (a) The PLC of bins of rings pooled across elevations at −1 MPa (circles) and −2 MPa (triangles). (b) The PLC of each elevation pooled across all of the bins at −1 MPa (circles) and −2 MPa (triangles). Uppercase letters represent significance at a water potential of −2 MPa, and lowercase letters represent significance at a water potential of −1 MPa.

We did not detect a significant difference in PLC at −1 MPa among the different elevations (*F*_2,77_ = 0.416, *P* = 0.6613). There was, however, a significant difference in PLC among the bins (*F*_3,77_ = 5.027, *P* = 0.0031). Pairwise comparisons confirmed that the rings older than 3 yr had more PLC at −1 MPa than the newest ring and the full cross‐sectional area (Fig. 2a). At −2 MPa, we found no significant differences among the elevations with data from all bins pooled together (*F*_2,78_ = 2.842, *P* = 0.0643) or among the bins of rings (*F*_3,78_ = 0.475, *P* = 0.7009; Fig. 2a,b), suggesting equally embolized vessels after −2 MPa. We found no significant difference in the *P*_50_ among the bins of rings (*F*_3,81_ = 0.5562, *P* = 0.6455) or elevations (*F*_2,82_ = 0.5398, *P* = 0.5849; Fig. S5). For results of analyses without the bin containing the entire cross‐sectional area, see Table S5. For vulnerability and conductivity decline curves from the elevational climatic gradient, see Fig. S6.

#### Dye perfusions among elevations

We found no significant differences in the active proportion of xylem per bin among the elevations ($\chi^{2}$ = 0.415, df = 2, *P* = 0.813). There were, however, differences in the active proportion of xylem in each bin of rings ($\chi^{2}$ = 43.888, df = 2, *P* < 0.001). The newest ring had a higher proportion of active xylem than the 2‐ and 3‐yr‐old rings and the rings older than 3 yr (Fig. S7).

### Cross‐population climatic gradient

#### Ring‐specific vulnerability curves among the populations

**Fig. 3:**
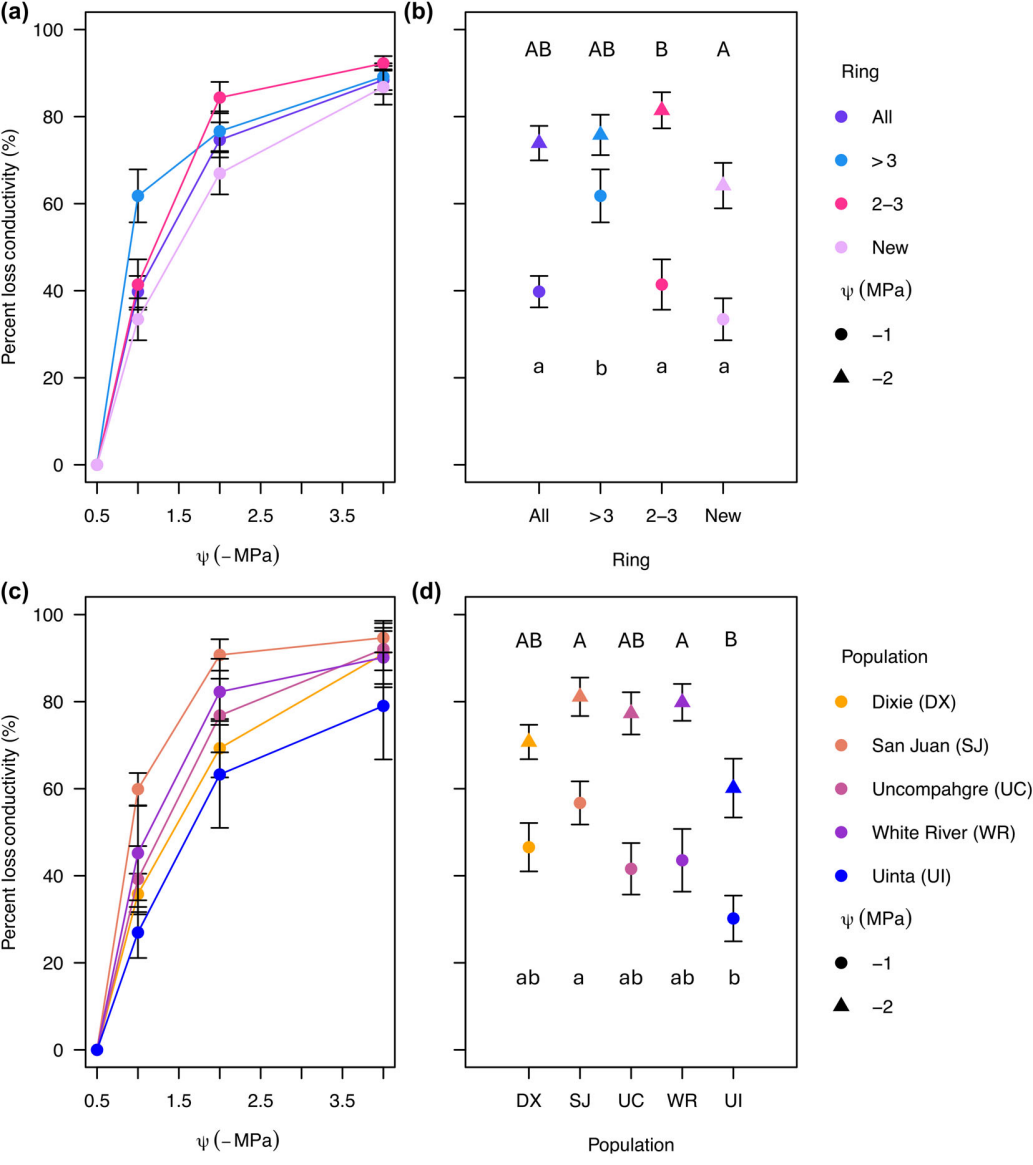
Trembling aspen vulnerability curves and percent loss in conductivity (PLC) at −1 and −2 MPa for the bins of rings and the different populations across the climatic gradient. (a)Vulnerability curves of the different bins of rings pooled across populations and (c) across the five different populations for the entire cross‐sectional area. Percent loss in conductivity at −1 MPa (circles) and at −2 MPa (triangles) for each bin of rings pooled across the populations (b) and for each population pooled across all of the bins (d). Points represent the mean and error bars represent the SE. Uppercase letters represent significance at −2 MPa, and lowercase letters represent significance at −1 MPa.

We built vulnerability curves for each bin of rings for each population (Fig. 3a,c). We found no significant differences among the populations in *P*_50_ when combining all bins (*F*_4,103_ = 1.2196, *P* = 0.3071). There were, however, differences among the bins (*F*_3,103_ = 2.7094, *P* = 0.0490), with the newest ring having a significantly more negative *P*_50_ than the rings older than 3 yr (*P* = 0.0443). We found significant differences among populations and bins at −1 MPa. The oldest rings (> 3 yr) had more PLC at −1 MPa than the entire cross‐sectional area, the 2‐ to 3‐yr‐old rings, and the newest ring (*F*_3,103_ = 5.746, *P* = 0.0011; Fig. 3b). There were significant differences among the populations at −1 MPa (*F*_4,103_ = 2.971, *P* = 0.0229; Fig. 3d), as San Juan had a higher PLC than Uinta (*P* = 0.0087). The PLC at −2 MPa differed in both the bins (*F*_3,110_ = 2.734, *P* = 0.0471; Fig. 3b) and the populations (*F*_4,110_ = 3.230, *P* = 0.0151; Fig. 3d). Two‐ and 3‐yr‐old rings had a significantly higher PLC at −2 MPa than the newest ring. Both San Juan and White River had a significantly higher PLC at −2 MPa than Uinta. For results of analyses without the bin containing the entire cross‐sectional area, see Table S6.

**Fig. 4:**
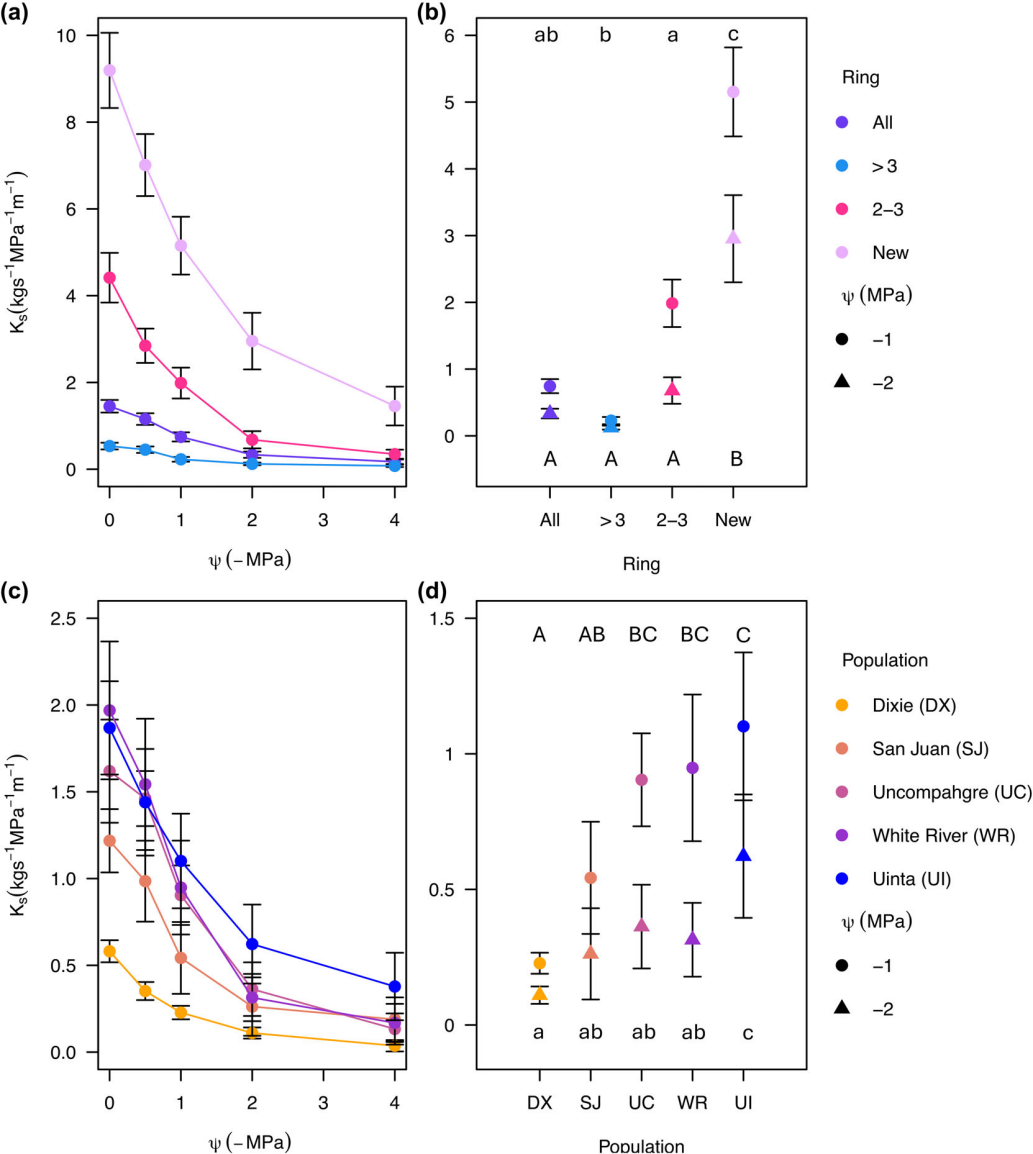
Xylem area‐specific conductivity (*K*_s_) decline curves of trembling aspen. Ks decline curves for the different bins of rings pooled across the populations (a) and for the five populations for the entire cross‐sectional area (c). The *K*_s_ of bins of rings at −1 MPa (circles) and at −2 MPa (triangles) for the different bins of rings pooled across the different populations (b) and for the different populations pooled across all bins of rings (d). Points represent the mean and error bars represent the SE. Uppercase letters represent significance at −2 MPa, and lowercase letters represent significance at −1 MPa.

We also built conductivity decline curves for each bin of rings for each population (Fig. 4a,c). For the *K*_s_ at −1 MPa, we found that the bins significantly differed from each other (*F*_3,112_ = 40.5669, *P* < 0.001). The newest ring had the highest *K*_s_ at −1, followed by all rings and 2‐ to 3‐yr‐old rings, with the oldest rings having the lowest *K*_s_ (Fig. 4b). At −1 MPa, some of the populations also significantly differed from each other (*F*_4,112_ = 7.1981, *P* < 0.001), with Uinta having more than two times higher *K*_s_ than the San Juans and more than four times higher *K*_s_ than Dixie (Fig. 4d). Dixie had a lower *K*_s_ than all populations except for San Juan. At −2 MPa, the bins did significantly differ (*F*_3,112_ = 15.8692, *P* < 0.001) with only the newest ring having a higher *K*_s_ than the other bins (Fig. 4b). The populations also differed (*F*_4,112_ = 3.7278, *P* = 0.0069) with Uinta having more than 7 times higher *K*_s_ than Dixie (Fig. 4d).

For *K*_max_, we detected a significant difference among the bins (*F*_3,112_ = 147.426, *P* < 0.001; Fig. S8a), where all of the rings significantly differed from each other. The newest ring had the highest conductivity per area, followed by 2‐ to 3‐yr‐old rings, then by all rings, and the rings older than 3 yr had the lowest conductivity per area. The populations also significantly differed from each other (*F*_4,112_ = 21.650, *P* < 0.001; Fig. S8b), with Uinta, White River, and Uncompahgre having the highest conductivity per area, San Juan in the middle, followed by Dixie having the lowest *K*_max_.

#### Vessel diameters

**Fig. 5:**
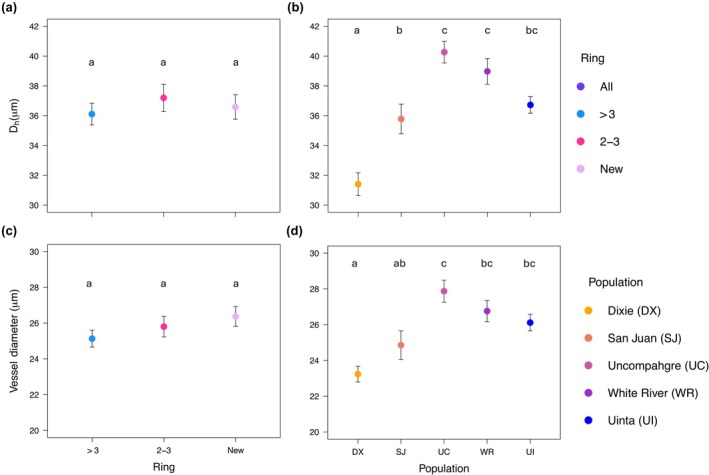
Mean hydraulic diameter (*D*_h_) and mean vessel diameter in trembling aspen. *D*_h_ of bins of rings pooled across populations (a) and for each population pooled across bins (b). Mean vessel diameter of the bins of rings pooled across populations (c) and the mean vessel diameters of the populations pooled across bins (d). Points represent the mean, and letters indicate significance while error bars represent the SE.

We found no significant difference in vessel diameters among the rings (*F*_2,83_ = 1.834, *P* = 0.166; Fig. 5c). There was, however, a significant difference in vessel diameter among the populations (*F*_4,83_ = 9.170, *P* < 0.001) with Uncompahgre having a significantly larger vessel diameter than Dixie and San Juan. All the other populations had larger vessel diameters than Dixie except for San Juan (Fig. 5d). For hydraulic diameter, there was also not a significant difference among the bins (*F*_2,83_ = 0.783, *P* = 0.461; Fig. 5a), but there was a difference among the populations (*F*_4,83_ = 18.371, *P* < 0.001). All populations had a larger hydraulic diameter than Dixie, followed by San Juan, and the other three populations did not significantly differ (Fig. 5b).

#### Dye perfusions

We did not detect a significant difference in the proportion of active xylem among the populations (χ^2^ = 5.456, df = 4, *P* = 0.244). The 2‐ to 3‐yr‐old rings and the newest ring did not significantly differ from each other, but the rings older than 3 yr had a significantly lower proportion of active xylem (χ^2^ = 38.463, df = 2, *P* < 0.001; Fig. S9).

#### Freezing days among populations and correlation with vessel diameter

There was a significant difference in the number of days below freezing per year among the populations (*F*_4,145_ = 39.137, *P* < 0.001). Dixie and Uinta National Forests had significantly more freezing days per year than San Juan, Uncompahgre, and White River National Forests. San Juan and White River National forests had the next highest number of days below freezing, and Uncompahgre had the least number of days below freezing (Fig. S10). There was a significant correlation between the average number of freezing days per year and the average vessel diameter, as the vessel diameter decreased with a greater number of freezing days per year (*S* = 180 526, ρ = −0.486, *P* < 0.001; Fig. S11).

### Additional methods validation

For the test looking at how much dye moved across ring boundaries, we found that an average of 1.95% of the glued xylem area had dye that leaked across the rings (Fig. S12). For the dye test comparing different conductivity states (flushed, native, or spun), there was no interaction among the independent variables. The low and high sites significantly differed from each other (*F*_1,105_ = 12.333; *P* < 0.001), with the highest site having more active xylem than the lower site (*P* < 0.001). The state that the stem went under dye perfusion (flushed, native, spun at −2 MPa) significantly differed (*F*_2,105_ = 3.211, *P* = 0.044), where stems that were flushed had significantly more active xylem area than those that were spun at −2 MPa. However, there were no differences between ‘flushed’ and ‘native’, nor were there differences between ‘native’ and the stems spun at −2 MPa. This indicates that the inactive xylem in the native state of the plant is likely due to permanent blockages, but at negative pressures, emboli are the cause for inactive xylem. The bins of rings significantly differed from each other (*F*_3,105_ = 66.631, *P* < 0.001), with the newest ring having significantly more percent xylem area stained, followed by the 2‐ and 3‐yr‐old rings, followed by the entire cross‐sectional area and then followed by the oldest rings (all *P*‐values < 0.001).

## Discussion

More frequent droughts due to climate change highlight an urgent need to better understand how multiple droughts and xylem aging affect plant water transport. Most vulnerability curves are constructed using the entire cross‐sectional area of a sample; thus, these curves do not account for differences among annual growth rings and may be overestimating the contribution of old rings to whole stem conductance (Melcher *et al*., 2003; Fukuda *et al*., 2015). In this study, we developed a novel method to quantify ring‐specific xylem function and found that rings of different ages differed in their ability to withstand embolism and conduct water. Using data from five *Populus tremuloides* populations growing in contrasting climatic regimes and three populations across an elevation/precipitation gradient, we found support for our first two hypotheses that older rings experienced not only lower area‐specific hydraulic conductivity but also increased vulnerability to embolism, indicating a decoupling of the safety vs efficiency trade‐off across individual rings. We also found support for our third hypothesis that particularly at drier sites and populations experienced more vulnerability and less conductivity. Taken together, these results indicate xylem that undergoes continued drought stress appears to accumulate damages and that the newest xylem that has not yet undergone continued drought stress contributes most to water transport and resistance to embolism.

### Ring‐specific contributions to drought tolerance and water transport

We found that the newest ring had a more resistant *P*_50_ than the rings older than 3 yr, and that the bins of rings reached different levels of PLC at −1 and − 2 MPa. There tended to be larger differences among the rings in this range of xylem tension (Fig. 3a), which is notable as this is a very typical summer midday water potential range for aspen in the SJNF (Anderegg *et al*., 2013b, L. D. L. Anderegg *et al*., 2013, Anderegg & Hillerislambers, 2016, Love *et al*., 2019; Fig. S3; for native PLC, see Fig. S13). Our results reveal that rings aged 1–3 yr are most resistant to emboli at these water potentials and that rings older than 3 yr are not able to safely operate under average summer conditions (PLC beyond *P*_50_ at average water potentials). These results are consistent with Pratt *et al*. (2020) in which they found that 1, 2‐, and 3‐yr‐old stems had good agreement in their benchtop dehydration curves. It is likely that the pit membranes from the older vessels are weakened because of cavitation fatigue, making the spread of emboli easier through ‘leaky pits’ (Sperry *et al*., 1991; Fukuda *et al*., 2015; Hillabrand *et al*., 2016). Additionally, it is also possible that these older rings are blocked due to the formation of tyloses. However, tyloses formation is frequently tied to embolism, xylem damage (Sperry *et al*., 1991; Taylor *et al*., 2002), wound response (Leśniewska *et al*., 2017), and we believe that drought stress and embolism from previous drought exposure plays a role in this process. By the time the entire cross‐sectional area had reached *P*_50_, the oldest rings were on average 67% embolized. Compared with other hardwoods, magnetic resonance imaging shows that by the time the whole stem had reached a 50% loss in conductivity, rings older than the current year were already 100% embolized (Fukuda *et al*., 2015). We generally found that the curve for the entire cross‐sectional area seemed to be an average between the curve of the newest ring and the rings older than 3 yr old and that the entire cross‐sectional area did not differ in PLC from the newest ring at −1 and −2 MPa. This is because the newest ring contributes the most to the conductivity and thus dominates the measurement signal. This finding has important methodological implications, as in some cases, it may not be necessary to perform the labor‐intensive process of performing ring‐specific vulnerability curves if PLC of the newest ring is desired, at least in this or similar species.

The old rings (> 3 yr) did not contribute much to the conductivity of the whole stem, and the way that *K*_s_ is typically measured, as area‐normalized by the whole stem, is likely an underestimation of the *K*_s_ of active xylem areas because of the inclusion of inactive xylem in the measurement. Our hypothesis that the newest ring would conduct more water and that old rings would conduct less water was supported by both the area of the dyed xylem and the *K*_s_. New rings, particularly the first third of the xylem from the cambium, are more efficient at water transport and contribute to half of the water transport of the stem in deciduous species (Melcher *et al*., 2003; Dang *et al*., 2014; Fukuda *et al*., 2015). This is likely because newer rings are responsible for water transport to the leaves (Melcher *et al*., 2003; Fukuda *et al*., 2015), while the rings older than 3 yr contain xylem that is less commonly used for water transport and is instead more often used for water storage (Tyree & Zimmermann, 2002). These patterns, however, are often clade‐ or species‐specific, and in the case of aspen, the leaves are supplied water by only its newest ring, and there is likely a very high resistance for water movement across the rings, but other studies have found pathways across growth rings (Pratt *et al*., 2020). We found that several other papers using a variety of methods across many species found similar trends that the radial connection across ring boundaries was minimal (Domec *et al*., 2005; Barnard *et al*., 2013; Wason *et al*., 2019; Petit *et al*., 2023).

While this method of doing vulnerability curves was generally successful in being able to tease apart differences in conductivity and vulnerability among annual growth rings, some caveats are worth noting. One is that vulnerability curves were not done with as high‐resolution water potential measurement points as is typically preferred. The lower resolution water potential bins were due to the need to cut the glue off before every spin in the centrifuge. Cutting the stem too often would render the stem too short for the rotor, so the V‐curve was abbreviated to include fewer spins. Additionally, we calculated the conductivity for 2‐ to 3‐yr‐old rings through the subtraction of the conductivity of the newest ring from the conductivity of the three newest rings. This way is likely not as accurate as directly measuring the conductivity of this bin, but was done to prevent more gluing and cutting.

### Variation across broad environmental gradients

When comparing among populations and across the elevational gradient, we did not find significant differences in the *P*_50_, but there were differences among populations at −1 and −2 MPa. San Juan had some of the most vulnerable xylem at −1 and −2 MPa, while Uinta had some of the most resistant xylem at these pressures. These results are similar to those found in Kerr *et al*. (2023) in which the drier populations were more vulnerable to embolism than the wetter population. This may indicate that dry populations (Dixie and San Juan NF) have experienced cavitation fatigue (Hacke *et al*., 2001). Extensive research has documented substantial drought‐driven aspen mortality in this region and supports the idea that these populations are undergoing hydraulic decline (Huang & Anderegg, 2012; Anderegg *et al*., 2013a,b).

As for the conductivity, Dixie had the lowest conductivity at −1 MPa, followed by the San Juans, with the other three populations not differing in conductivity. The differences in conductivity among the populations can be partially explained by the vessel diameter size, as larger vessels tend to be more hydraulically conductive (Sperry *et al*., 2006). Interesting future steps would include teasing apart if differences in *K*_h_ were mediated by vessel diameter or through the quantity of active xylem on the same stem. Additionally, we note the correlation between the vessel diameter size and the number of days below freezing (Fig. S10), which has been observed in other species (Davis *et al*., 1999). Both the CWD and the high number of days below freezing show that Dixie National Forest may experience both drought stress and freeze–thaw stress (Ewers *et al*., 2003), which can interact to create additional xylem stress (Langan *et al*., 1997; Feng *et al*., 2015). While hydraulic conductivity and vessel diameter were thought to correlate with water availability, vessel diameter was instead constrained by freeze–thaw stress and sites that were more mid‐range were more balanced between drought stress and freeze–thaw stress.

### Conclusion

In conclusion, our findings reveal that there are substantial differences in rings of varying ages to conduct water and resist emboli. In general, the newest ring was the most resistant at standard summer water potentials and contributed the most to water conductance, while the rings older than 3 yr were very vulnerable and did not contribute much to conductivity. These patterns were seen across broad scales in an elevation gradient and across a climatic gradient. Across a climatic gradient, populations experiencing the most drought stress were more vulnerable, highlighting an accumulation of damage signals rather than an acclimation signal. Additionally, the interaction between drought stress and freeze–thaw may have had impacts on conductivity and vessel diameter size. A broader understanding of how changes in ring‐specific water transport influence whole‐plant water fluxes and survival, as well as the changes in conductivity across rings in different taxa, is urgently needed to illuminate how more climate extremes will affect forests in coming decades.

## Competing interests

None declared.

## Author contributions

JCF and WRLA designed the experiment. JCF and GVG carried out the data collection and analyses. JCF led the writing of the manuscript. All authors interpreted the data, contributed to drafts, and gave final approval for publication.

## Disclaimer

The New Phytologist Foundation remains neutral with regard to jurisdictional claims in maps and in any institutional affiliations.

## Supporting information


**Fig. S1** Schematic detailing how ring‐specific hydraulic conductivity was measured and how the conductivity of each bin was calculated.
**Fig. S2** Climatic Water Deficit and Palmer Severity Drought Index data for each population along the climate gradient for years 2000–2022.
**Fig. S3** Water potential data for the San Juan National Forest.
**Fig. S4** Pressure at 50% loss of conductivity for the bins for the five different populations along the climatic gradient.
**Fig. S5** Pressure at 50% loss of conductivity for the bins for the sites along the elevational gradient.
**Fig. S6** Vulnerability curves and conductivity decline curves for the sites along the elevational gradient.
**Fig. S7** Proportion of active xylem for each bin across the elevational gradient.
**Fig. S8** Maximum conductivity for each of the bins and for each of the populations along the climatic gradient.
**Fig. S9** Proportion of active xylem for each bin across the climatic gradient.
**Fig. S10** Number of days below freezing for the different populations across the climatic gradient.
**Fig. S11** Correlation between the number of days below freezing per year and the vessel diameter for each of the populations along the climatic gradient.
**Fig. S12** Percentage of xylem area that experienced dye leakage past the glued‐off area.
**Fig. S13** Native Percent Loss in Conductivity measured for the different populations across the climatic gradient.
**Table S1** Seasonal temperature and precipitation data for sites along the San Juan elevation gradient.
**Table S2** Latitude, longitude, and elevation data for sites along the San Juan elevation gradient.
**Table S3** Seasonal temperature and precipitation data for the five different populations along the climatic gradient.
**Table S4** Climatic Water Deficit maximum and Palmer Severity Drought Index minimum for 3 drought years data for the five different populations along the climatic gradient.
**Table S5** Additional analyses of *P*_50_ and PLC data with the bin containing the entire cross‐sectional area removed for the elevational gradient.
**Table S6** Additional analyses of *P*_50_ and PLC data with the bin containing the entire cross‐sectional area removed for the climatic gradient.
Please note: Wiley is not responsible for the content or functionality of any Supporting Information supplied by the authors. Any queries (other than missing material) should be directed to the *New Phytologist* Central Office.

## Data Availability

The hydraulic and trait data, as well as the code for analysis, can be found at the following figshare repository doi: 10.6084/m9.figshare.28670264.
