# Supplementary material for: Ring‐specific vulnerability to embolism reveals accumulation of damage in the xylem
Source: New Phytol. 2025 Apr 18;246(5):2046–58. doi: 10.1111/nph.70137 (PMC12059512; doi:10.1111/nph.70137)
Supplement: Supplementary file 1 — Fig. S1 Schematic detailing how ring‐specific hydraulic conductivity was measured and how the conductivity of each bin was calculated. Fig. S2 Climatic Water Deficit and Palmer Severity Drought Index data for each population along the climate gradient for years 2000–2022. Fig. S3 Water potential data for the San Juan National Forest. Fig. S4 Pressure at 50% loss of conductivity for the bins for the five different populations along the climatic gradient. Fig. S5 Pressure at 50% loss of conductivity for the bins for the sites along the elevational gradient. Fig. S6 Vulnerability curves and conductivity decline curves for the sites along the elevational gradient. Fig. S7 Proportion of active xylem for each bin across the elevational gradient. Fig. S8 Maximum conductivity for each of the bins and for each of the populations along the climatic gradient. Fig. S9 Proportion of active xylem for each bin across the climatic gradient. Fig. S10 Number of days below freezing for the different populations across the climatic gradient. Fig. S11 Correlation between the number of days below freezing per year and the vessel diameter for each of the populations along the climatic gradient. Fig. S12 Percentage of xylem area that experienced dye leakage past the glued‐off area. Fig. S13 Native Percent Loss in Conductivity measured for the different populations across the climatic gradient. Table S1 Seasonal temperature and precipitation data for sites along the San Juan elevation gradient. Table S2 Latitude, longitude, and elevation data for sites along the San Juan elevation gradient. Table S3 Seasonal temperature and precipitation data for the five different populations along the climatic gradient. Table S4 Climatic Water Deficit maximum and Palmer Severity Drought Index minimum for 3 drought years data for the five different populations along the climatic gradient. Table S5 Additional analyses of P 50 and PLC data with the bin containing the entire cross‐sectional area removed for t [file NPH-246-2046-s001.docx]

*New Phytologist* Supporting Information

Article title: Ring-specific vulnerability to embolism reveals accumulation of damage in the xylem

Authors: Jaycie C. Fickle, German Vargas G. and William R.L. Anderegg

Article acceptance date: 17 March 2025.

**Table S1**. Seasonal temperature and precipitation data for the sites along the San Juan National Forest elevational transect as estimated by PRISM. Data represent 30-year normal from 1991 –2020 (accessed 19 December, 2024). Seasons are described as spring (March-May), summer (June- August), fall (September-November), and winter (December-February).

|  | Season | Low | Mid | High |
| --- | --- | --- | --- | --- |
| Max Temp (°C) | Spring | 11.23 | 10.27 | 9.93 |
|  | Summer | 23.30 | 22.23 | 21.83 |
|  | Fall | 13.40 | 12.43 | 12.17 |
|  | Winter | 2.73 | 1.97 | 1.90 |
| Min Temp (°C) | Spring | -3.43 | -3.33 | -4.27 |
|  | Summer | 7.27 | 7.77 | 6.60 |
|  | Fall | -1.00 | -0.53 | -1.53 |
|  | Winter | -10.90 | -10.37 | -11.37 |
| Precipitation (mm) | Spring | 50.56 | 55.69 | 55.77 |
|  | Summer | 49.71 | 55.09 | 60.04 |
|  | Fall | 56.18 | 60.22 | 61.61 |
|  | Winter | 62.53 | 68.36 | 70.85 |

**Table S2**. Latitude, longitude, and elevation (in meters) for each of the sites along the San Juan National Forest elevational transect. Elevation data was obtained from the USGS Elevation Point Query Service (U.S. Geological Survey 2023).

| Site | Latitude | Longitude | Elevation (m) |
| --- | --- | --- | --- |
| Low | 37.484 | -108.252 | 2664.033 |
| Mid | 37.482 | -108.194 | 2864.624 |
| High | 37.474 | -108.154 | 3074.281 |

**Table S3**. Seasonal climate data for each adult aspen population as estimated by PRISM. Data represent 30-year normals from 1991-2020. Seasons are described as spring (March-May), summer (June- August), fall (September-November), and winter (December-February). Sites are ranked from warm and dry to cool and wet going from left to right.

|  | Season | Dixie | San Juan | Uncompahgre | White River | Uinta |
| --- | --- | --- | --- | --- | --- | --- |
| Max Temp (°C) | Spring | 9.47 | 10.27 | 11.2 | 9.93 | 7.93 |
|  | Summer | 21.77 | 22.23 | 24.1 | 22.83 | 21.67 |
|  | Fall | 12.23 | 12.43 | 13.23 | 12.03 | 10.4 |
|  | Winter | 2.4 | 1.97 | 2.30 | 0.73 | -0.77 |
| Min Temp (°C) | Spring | -4.33 | -3.33 | -2.7 | -3.57 | -4.93 |
|  | Summer | 6.6 | 7.77 | 9.13 | 7.57 | 6.87 |
|  | Fall | -1.67 | -0.53 | 0.13 | -1.1 | -1.8 |
|  | Winter | -10.43 | -10.37 | -9.67 | -11.17 | -11.03 |
| Precipitation (mm) | Spring | 49.24 | 55.69 | 71.28 | 65.94 | 78.94 |
|  | Summer | 41.76 | 55.09 | 34.94 | 43.54 | 37.7 |
|  | Fall | 49.12 | 60.22 | 59.03 | 64.47 | 62.95 |
|  | Winter | 62.98 | 68.36 | 94.82 | 70.60 | 85.87 |

**Table S4**. The maximum Climatic Water Deficit (CWD; mm H_2_O) and minimum Palmer Drought Severity Index (PDSI) for the years 2018, 2020, and 2021 which were severe drought years (below -4 PDSI) for each population.

|  | 2018 | | 2020 | | 2021 | |
| --- | --- | --- | --- | --- | --- | --- |
|  | CWD Max | PDSI Min | CWD Max | PDSI Min | CWD Max | PDSI Min |
| Dixie | 172.9 | -4.3 | 192.3 | -5.9 | 186.1 | -7 |
| San Juan | 138.5 | -5.8 | 147.9 | -6.5 | 132.3 | -6.2 |
| Uncompahgre | 171.9 | -5.8 | 173.9 | -7.2 | 164.2 | -6.9 |
| White River | 155.4 | -5.7 | 159.4 | -7.1 | 153.1 | -7 |
| Uinta | 145.0 | -4.7 | 161.9 | -5.3 | 128.8 | -5.2 |

**Table S5.** Additional analyses of the Pressure at 50% loss in conductivity (P_50_) and Percent Loss Conductivity (PLC) of aspen at -1 and -2 MPa comparing across the bins for the elevational gradient including the bin that contains all of the rings “All” and with that bin removed.

|  | Including “All” bin | Excluding “All” bin |
| --- | --- | --- |
| P_50_ cutoff at -4 MPa | F_3,81_ = 0.5562, P = 0.646 | F_2,55_ = 0.402, P = 0.6710 |
| P_50_ cutoff at -3.5 MPa | F_3,79_ = 0.4050, P = 0.750 | F_2,54_ = 0.3439, P = 0.7106 |
| PLC at -1 MPa | F_3,77_ = 5.027, P = 0.003 | F_2,47_ = 4.338, P = 0.018 |
| PLC at -2 MPa | F_3,78_ = 0.475, P = 0.701 | F_2,52_ = 0.072, P = 0.93 |

**Table S6.** Additional analyses of the Pressure at 50% loss in conductivity (P_50_) and Percent Loss of Conductivity (PLC) of aspen at -1 and -2 MPa comparing across the bins for the climatic gradient including the bin that contains all of the rings “All” and with that bin removed.

|  | Including “All” bin | Excluding “All” bin |
| --- | --- | --- |
| P_50_ cutoff at -4 MPa | F_3,103_ = 2.709, P = 0.049 | F_2,75_ = 4.077, P = 0.021 |
| P_50_ cutoff at -3.5 MPa | F_3,100_ = 1.667, P = 0.179 | F_2,73_ = 2.414, P = 0.097 |
| PLC at -1 MPa | F_3,103_ = 5.746, P = 0.001 | F_2,74_ = 6.912, P = 0.002 |
| PLC at -2 MPa | F_3,110_ = 2.733, P = 0.047 | F_2,80_ = 3.856, P = 0.025 |


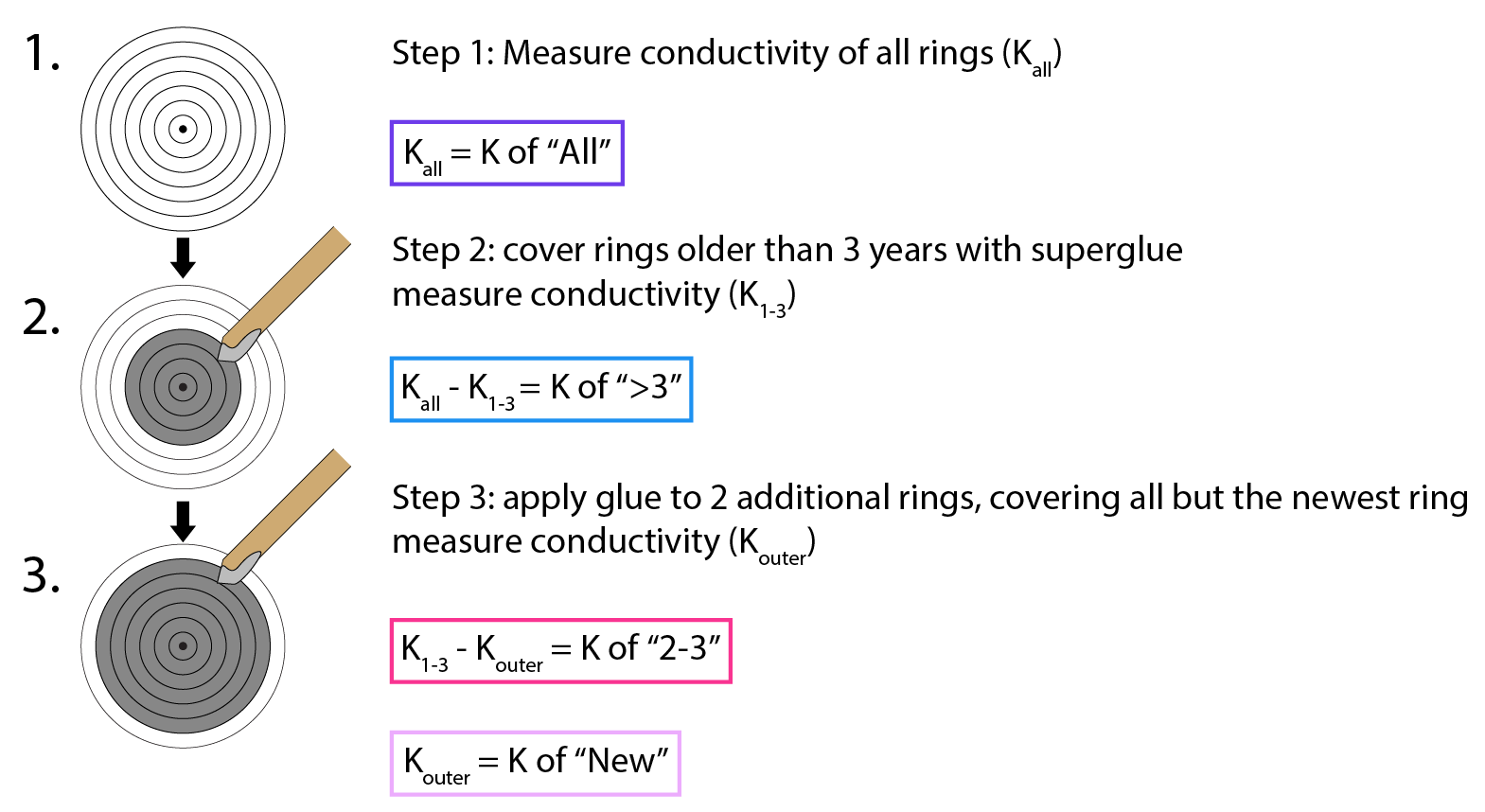


**Figure S1**. Stepwise schematic detailing how ring-specific hydraulic conductivity was measured and how the conductivity (K) of each bin of rings was calculated.

**Figure S2**. a) Climatic Water Deficit (CWD) (a) and Palmer Drought Severity Index (b) for each of the adult aspen populations from 2000 – 2022. The solid line marks a PDSI of 0, where everything above 0 is classified as “Wet” and everything below 0 is classified as “Drought”. A PDSI of -4 is indicated with a dashed line and anything below that line is considered severe drought.

**Figure S3.** Operating xylem tension for trembling aspen in the San Juan National Forest Predawn and Midday water potentials ($\psi$_leaf_) as measured from the San Juan elevation gradient aspen stands during the summer drought. Data from Anderegg and Hilleslambers (2016) and Kerr et al. (2023). The horizontal black line represents the median, the colored bars represent the upper and lower quartile, while the vertical black lines represent the maximum and minimum values. Transparent boxes represent the predawn water potential while saturated boxes represent the midday water potential.


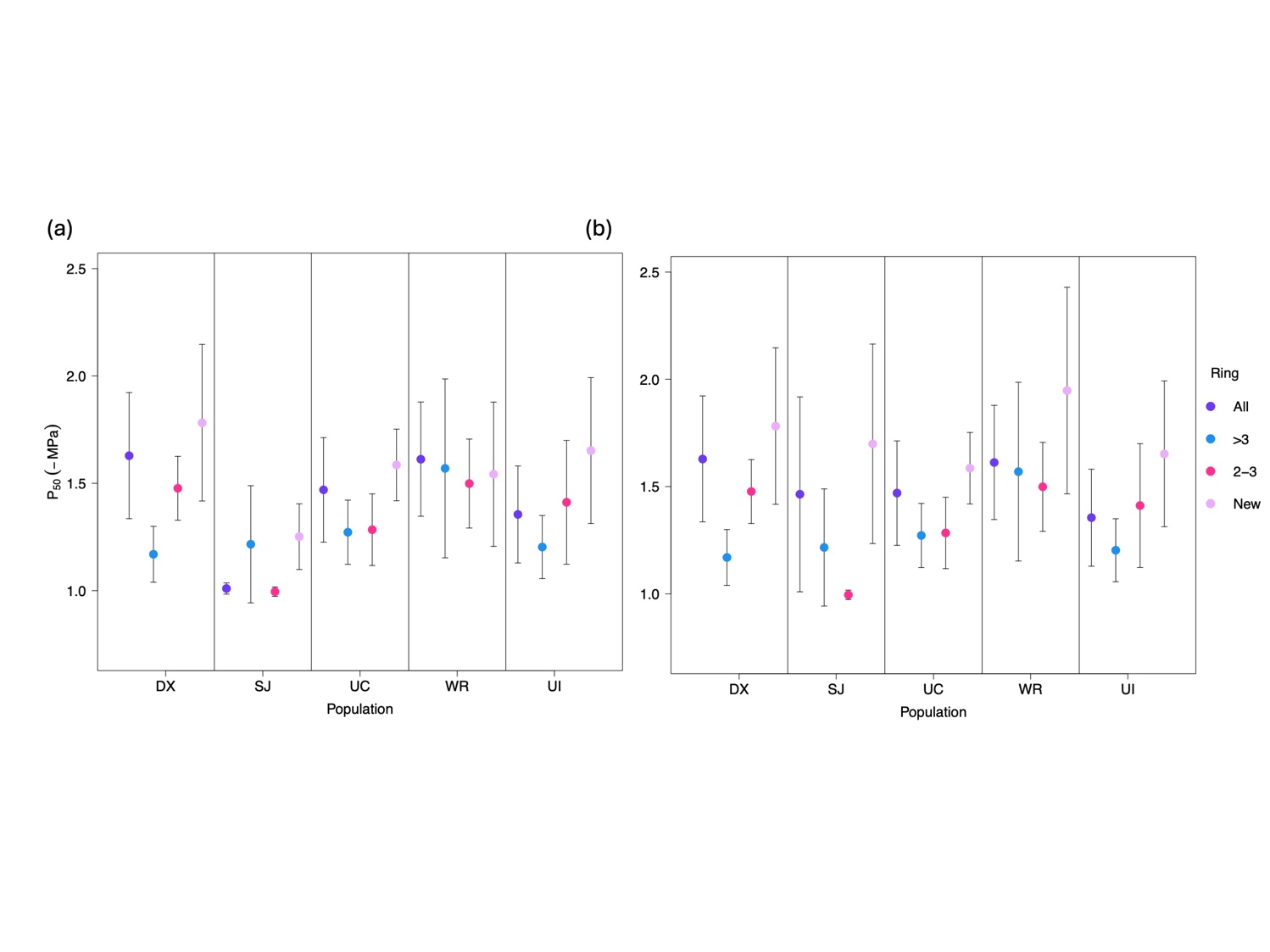


**Figure S4.** The Pressure at 50% loss in hydraulic conductivity (P_50_) values of trembling aspen for each Bin across the 5 populations. The error bars represent the standard error and the colored dots represent the mean. a) Plot of P_50_ with a cutoff at -3.5 for outliers. b) Plot of P_50_ with a cutoff at -4 for outliers.


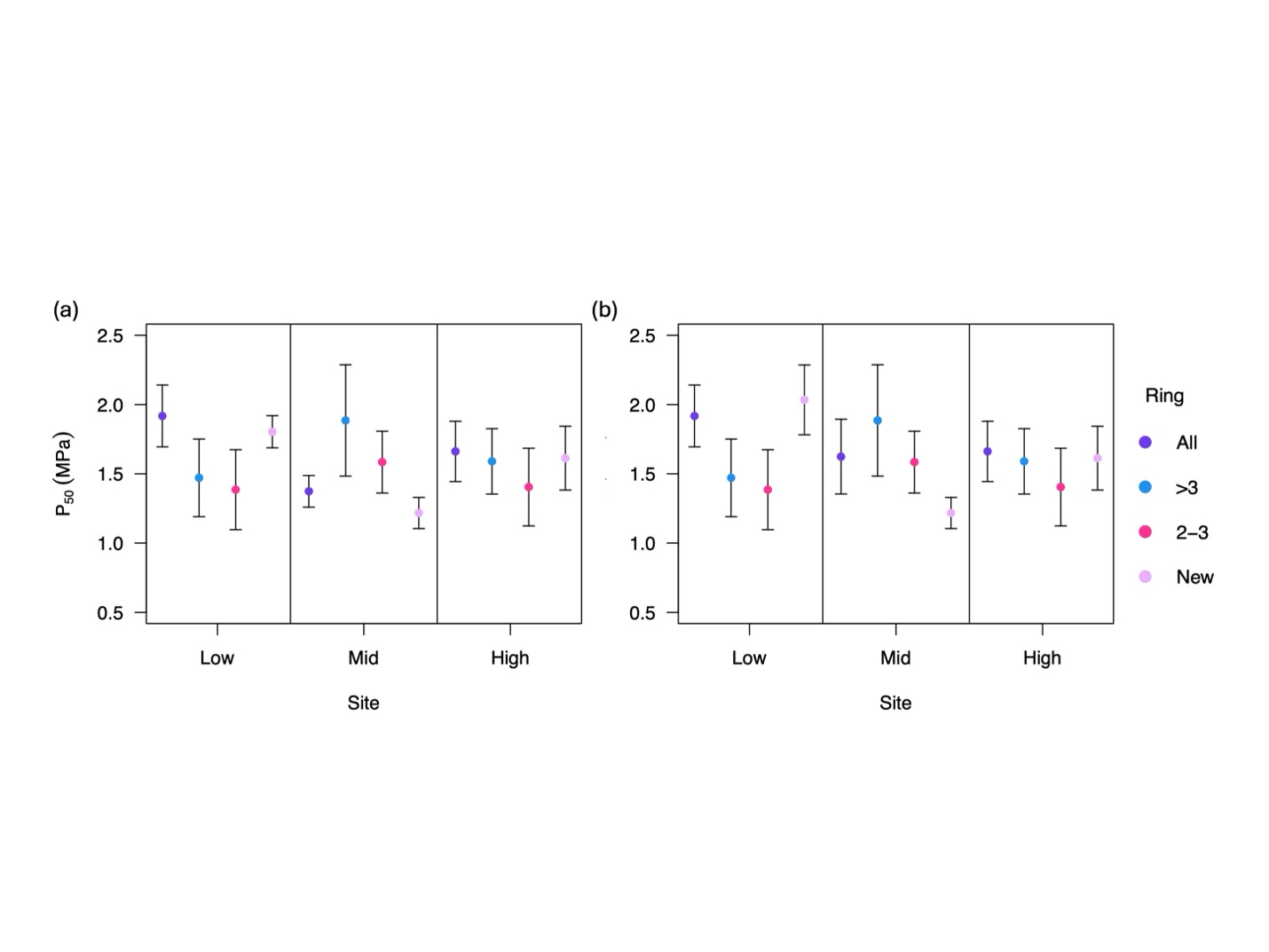


**Figure S5.** The Pressure at 50% loss in hydraulic conductivity (P_50_) values of trembling aspen for each Bin across the 3 elevation sites. The error bars represent the standard error and the colored dots represent the mean. a) Plot of P_50_ with a cutoff at -3.5 for outliers. b) Plot of P_50_ with a cutoff at -4 for outliers.


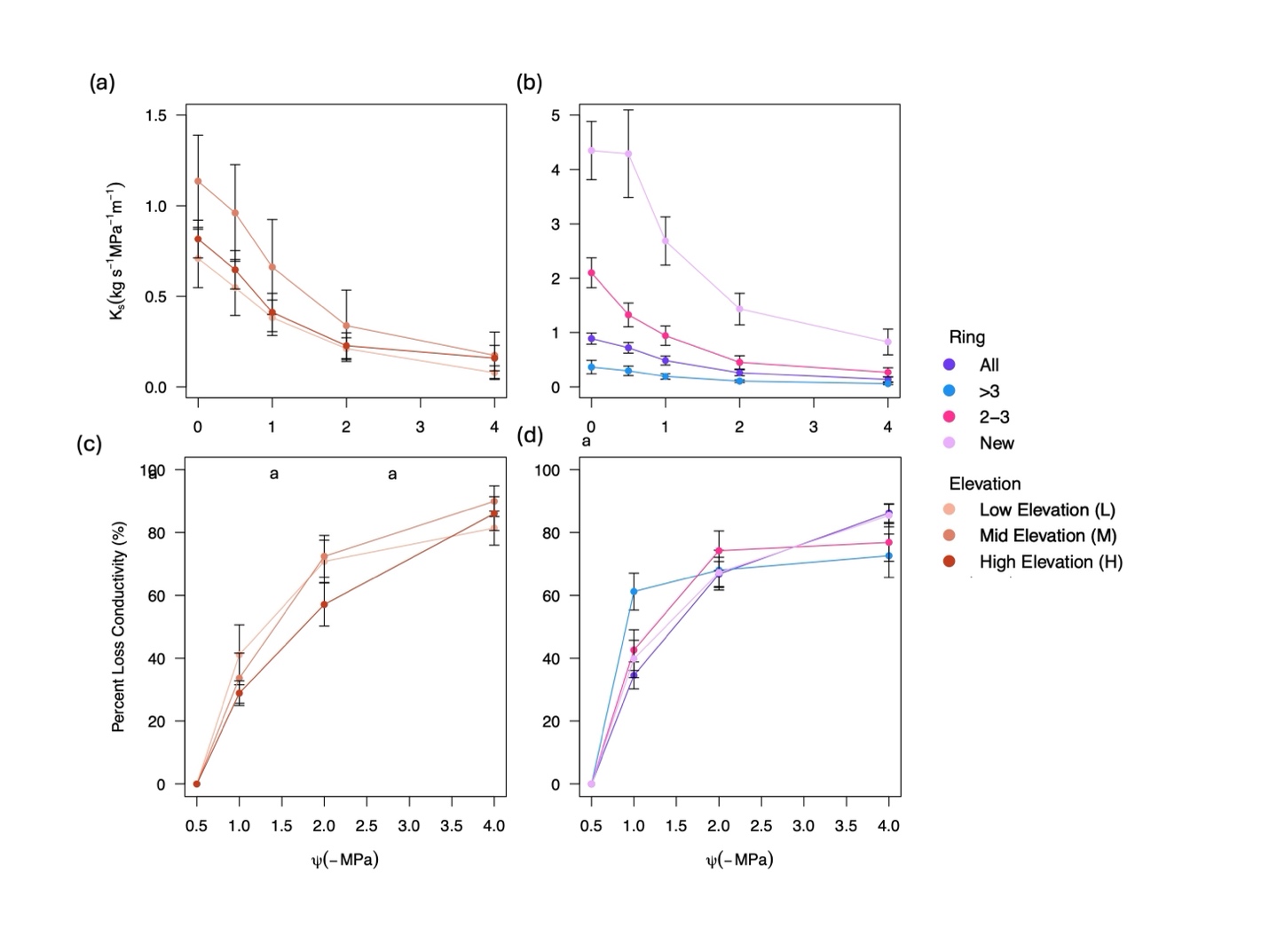


**Figure S6**. Xylem area specific conductivity (Ks) decline curves data and Vulnerability curves of trembling aspen. Ks decline curves for the three different elevational sites for the entire cross-sectional (a) and for the for different bins of rings pooled across the sites (b). Vulnerability curves of the three elevational sites for the entire cross-sectional area (c) and for the different bins of rings pooled across the sites (d). Points represent the mean and error bars represent the standard error.


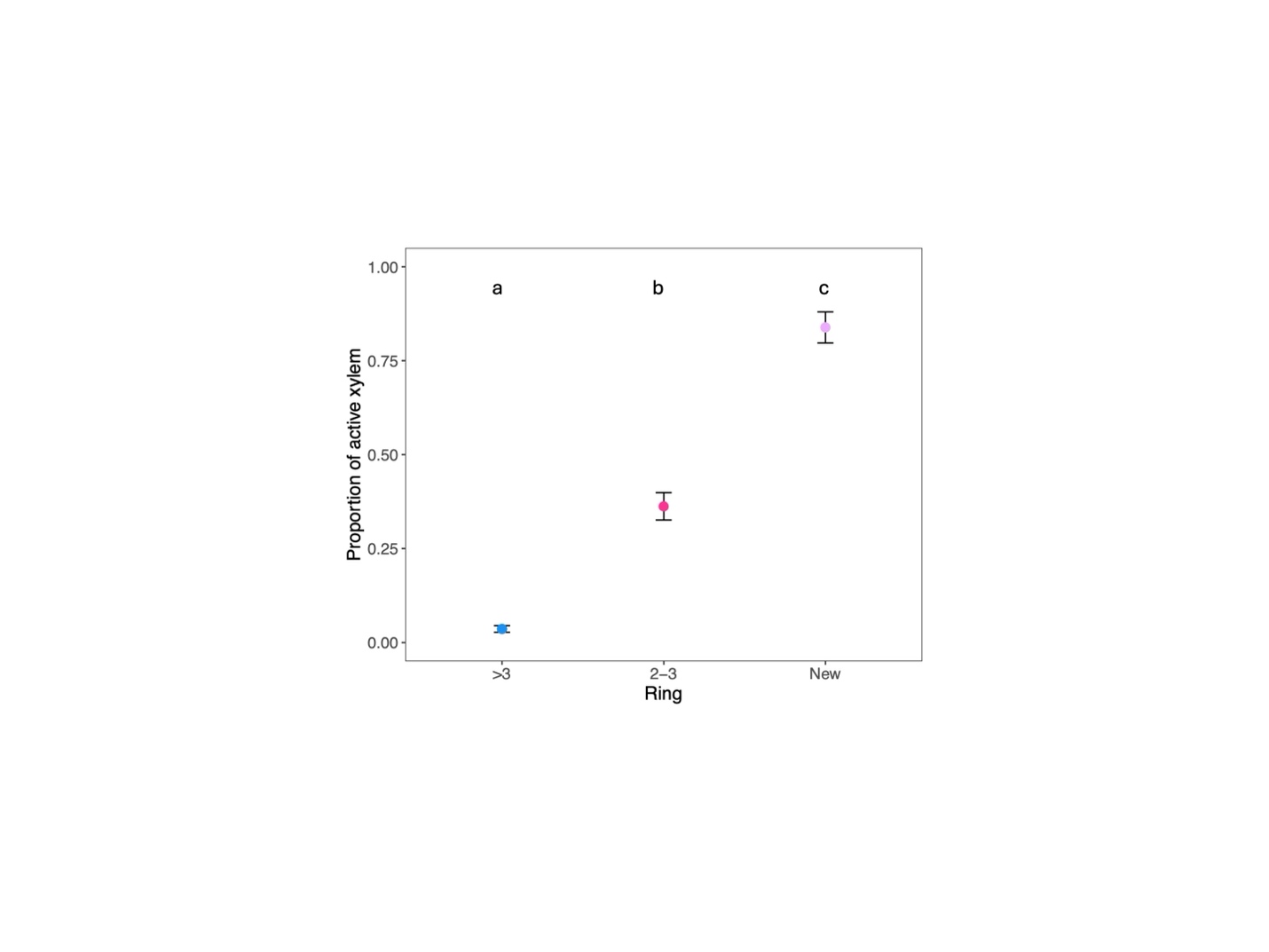


**Figure S7.** The proportion of active xylem measured from dye perfusions of trembling aspen for each bin of rings pooled across the different elevations of the elevational climatic gradient. Colored dots represent the mean and the error bars represent the standard error. Lowercase letters signify significant differences.


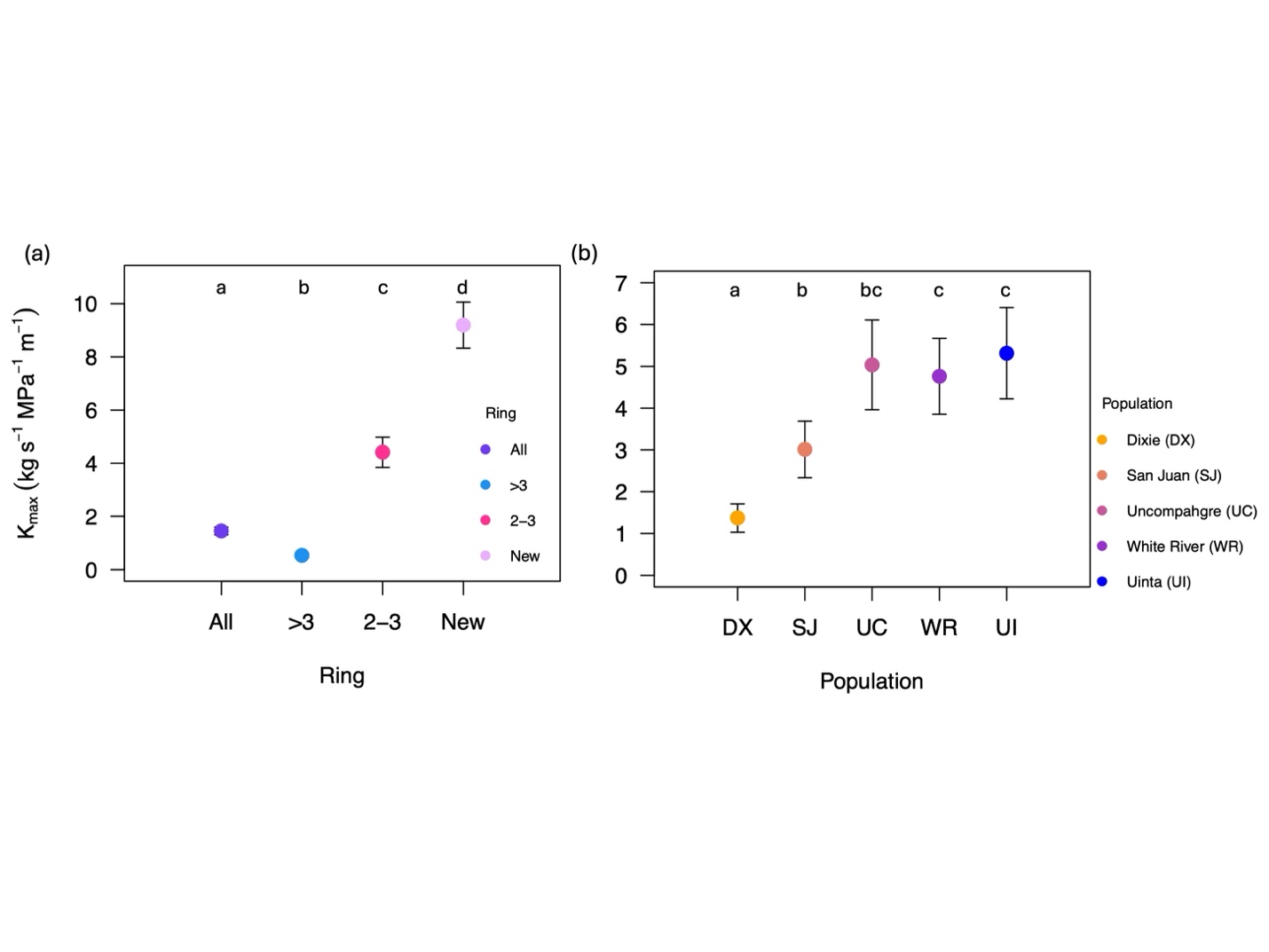


**Figure S8.** Maximum conductivity (K_max_) per area of trembling aspen where the point is the mean, and the error bars represent the standard error. (a) Comparing among the different bins. Bins “All” and “>3” do contain error bars, but are too small to show on the graph. (b) Comparing K_max_ among the different populations.


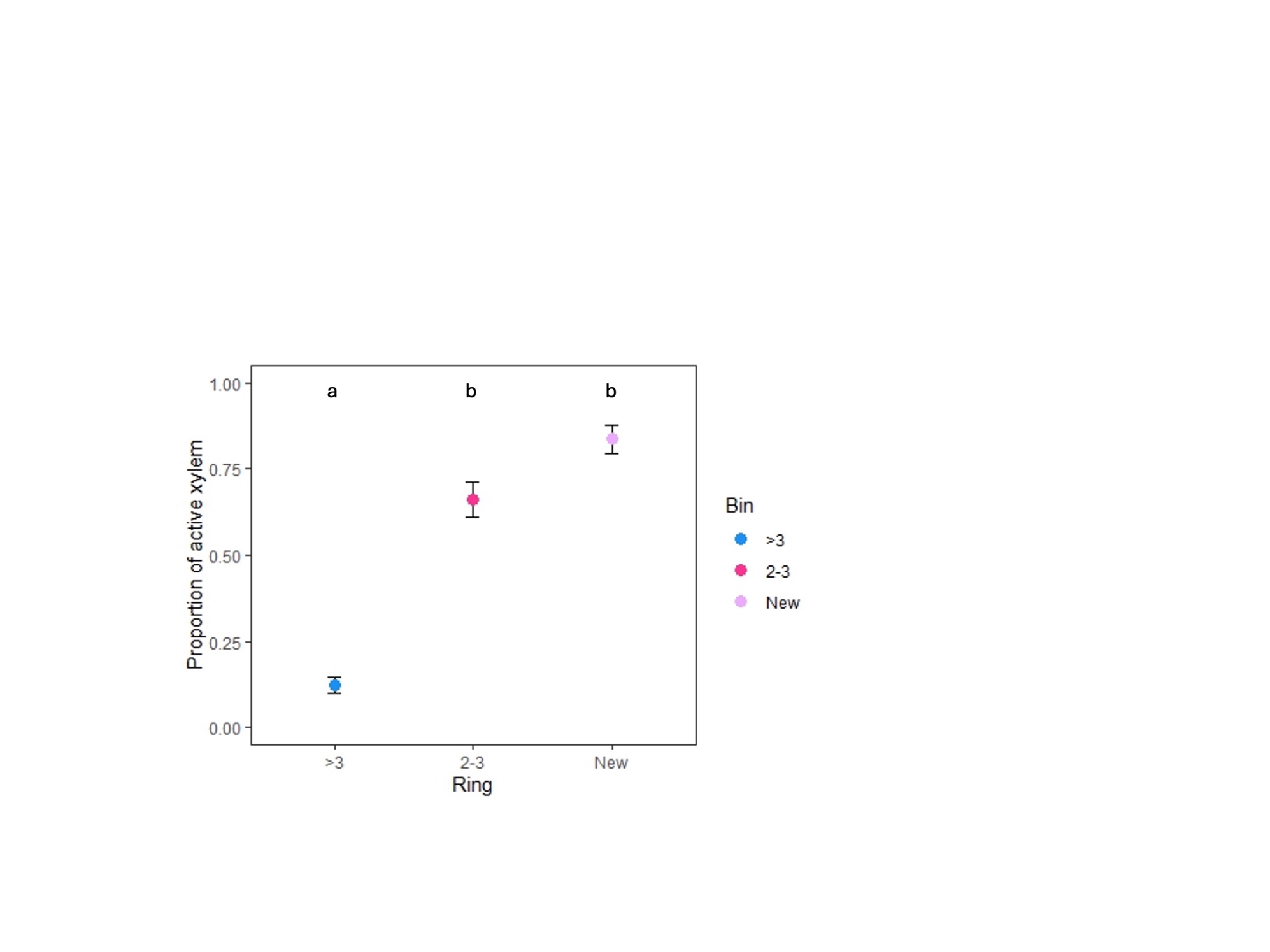


**Figure S9.** Proportion of active xylem of trembling aspen for each bin of rings pooled across populations as measured by dye perfusions. The colored dots represent the means and the error bars represent the standard errors. Lowercase letters indicate significance.


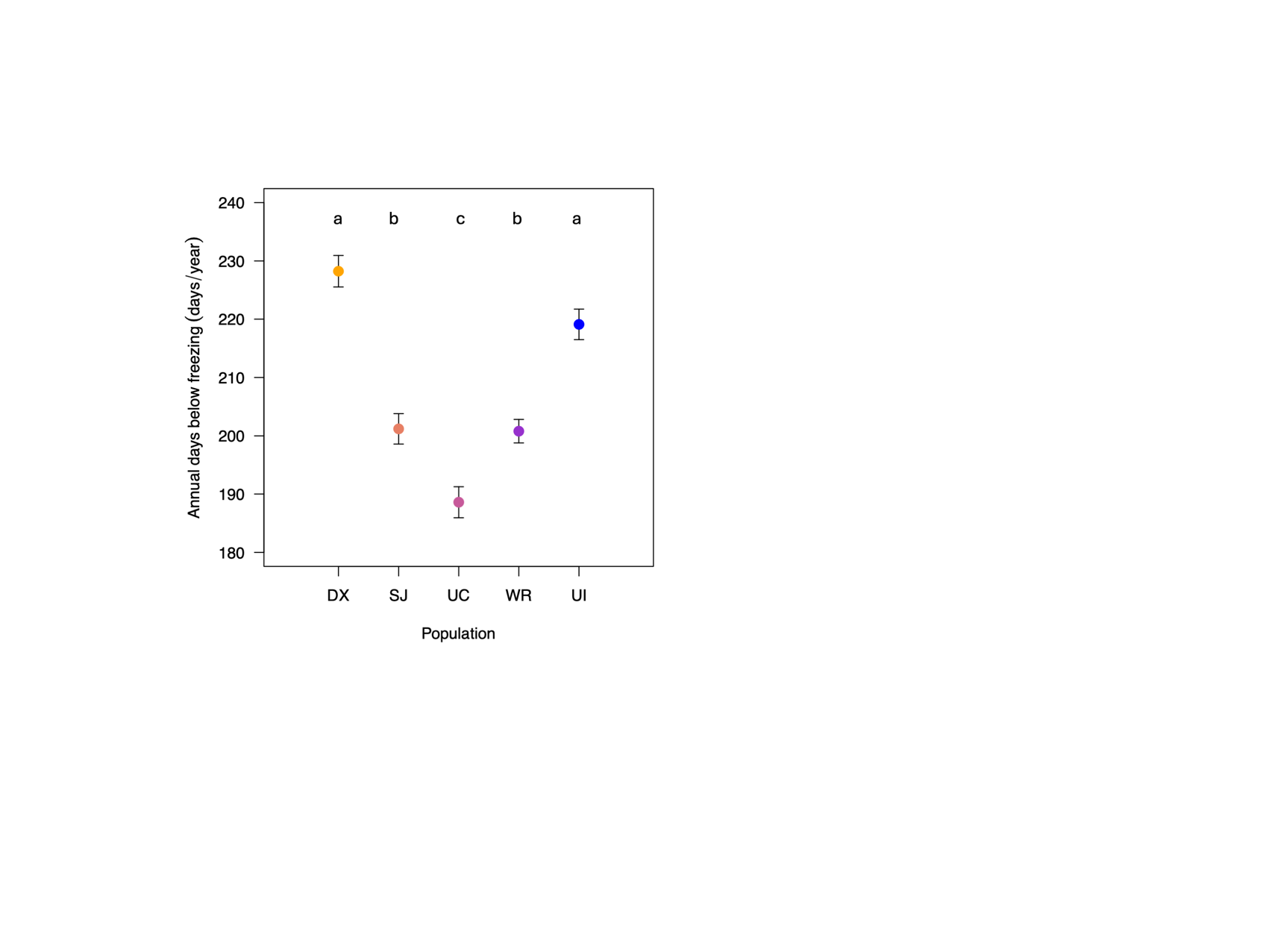


**Figure S10.** The average number of days below freezing between 1991-2020 for each adult aspen population. The populations are as follows: Dixie National Forest (DX), San Juan National Forest (SJ), Uncompahgre National Forest (UC), White River National Forest (WR), and Uinta National Forest (UI).


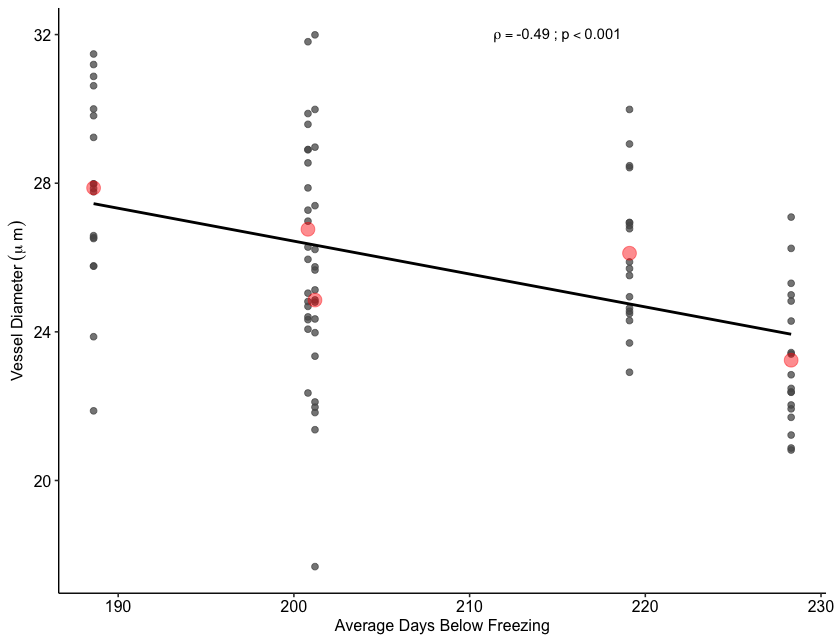


**Figure S11.** Correlation between average vessel diameter and the average days below freezing among the five adult aspen populations along the climatic gradient. Red dots represent the average vessel diameter, while the grey dots represent the spread of vessel diameters for each population.

**Figure S12**. Histogram of the percentage of area of xylem that experienced dye leakage past the area that was glued off with superglue in trembling aspen.

**Figure S13.** Native Percent Loss Conductivity for the 5 adult aspen populations. The populations are as follows: Dixie National Forest (DX), San Juan National Forest (SJ), Uncompahgre National Forest (UC), White River National Forest (WR), and Uinta National Forest (UI). The colored dot represents the mean and the error bars represent the standard error.

References:

**Anderegg, LDL, Hillerislambers, J.** 2016. Drought stress limits the geographic ranges of two tree species via different physiological mechanisms. *Global Change Biology* **22**: 1029-1045.

**Kerr KL, Fickle JC, Anderegg WRL. 2023.** Limited role of functional traits in explaining aspen growth and drought stress tolerance. *New Phytologist*: <https://doi.org/10.1111/nph.18937>.

**US.** Geological Survey. 2023. Elevation Point Query Service. https://apps.nationalmap.gov/epqs/.
